# High responsivity and 1/*f* noise of an ultraviolet photodetector based on Ni doped ZnO nanoparticles

**DOI:** 10.1039/c8ra05567j

**Published:** 2018-09-19

**Authors:** Imen Ben Elkamel, Nejeh Hamdaoui, Amine Mezni, Ridha Ajjel, Lotfi Beji

**Affiliations:** Laboratoire des Energies et des Matériaux, LabEM-LR11ES34, Ecole Supérieure des Sciences et de la Technologie, Université de Sousse Rue Lamine Abessi 4011, Hammam Sousse Tunisia Imenbenelkamel92@yahoo.com; Institut Supérieur des Technologies de l'Informatique et de la Communication, Université de Sousse Gp1, 4011 Hammam Sousse Tunisia; Unite de Recherche “Synthèse et Structure de Nanomatériaux” UR11ES30, Faculté des Sciences de Bizerte, Université de Carthage 7021 Jarzouna Tunisia

## Abstract

This study involves the novel fabrication of a high responsivity, fast response, and low-cost (UV) photodetector (PD) based on ZnO/Ni nanoparticles deposited on a glass substrate. The ZnO/Ni nanoparticles were synthesized using a polyol process. The structure and the morphology of the samples were characterized by X-ray diffraction (XRD) and Transmission Electron Microscopy (TEM). Optical properties were measured using UV-visible, diffuse reflectance and photoluminescence (PL) spectroscopy. The photodetector exhibited high photoresponse characteristics under 375 nm laser excitation. Our device shows a high responsivity (121 A W^−1^) with rise time (about 5.52 s) and fall time (about 12 s) at a bias voltage of 1 V. The device exhibits excellent reproducibility and stability characteristics with time. The noise spectra obtained from the UV photodetector were caused by the 1/*f* noise. The noise-equivalent power (NEP) is 1.08 × 10^−9^ W. Thus, the polyol process can be a useful and effective method for improving the performance of ZnO/Ni UV photodetectors.

## Abstract 2

This study involves the novel fabrication of a high responsivity, fast response, and low-cost (UV) photodetector (PD) based on ZnO/Ni nanoparticles deposited on a glass substrate.

## Graphical abstract

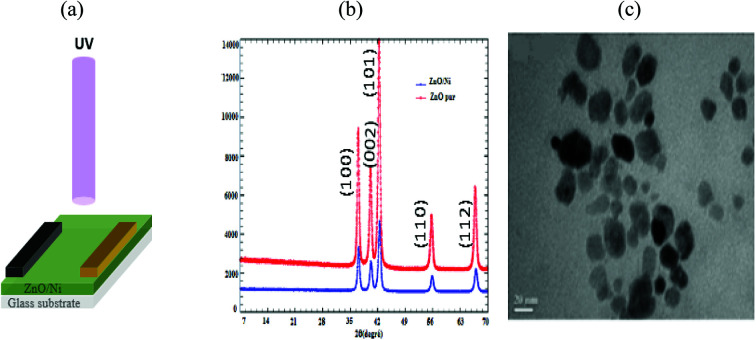

## 1 Introduction

Ultraviolet photodetectors (UV PDs) are widely used in the military and civil fields, for example in flame and radiation detection, optical communications, and binary switches.^1–4^ In the last decade, UV detectors based on wide band gap semiconductors (such as SiC, GaN, TiO_2_ and ZnO^5–9^) have received a lot of attention. Among the wide-band gap semiconductors, ZnO has many unique properties such as higher saturated carrier drift rate, wide band-gap and low cost.^10–12^ Meanwhile, ZnO has been regarded as one as the most suitable materials for the fabrication of high performance UV PDs.

Until now, ZnO-based UV photodetectors with different device structures have been investigated, such as Schottky junction, p–n junction and metal–semiconductor–metal (MSM) structure.^13–16^ According to the previous reports, the performance of these devices is generally dependent on the elaboration method of ZnO-based semiconductor nanomaterials. Additionally, ZnO demonstrates a large adsorption/desorption behavior of oxygen on the surface, which could decrease the dark current and increase the responsivity of the ZnO-based UV photodetectors.^14^ However, these adsorption/desorption processes of oxygen on the surface of ZnO always contribute to a long rise/decay time of ZnO UV PDs, which limited their practical applications.^15^ To overcome this drawback, some properties of ZnO, like optical and electrical properties, should be practically changed by use of some dopant elements for adjusting the band gap of ZnO.^2^ Doping of Ni into ZnO nanostructure for photodetector application enhances the sensitivity of the photodetector. Because the valence of Zn^2+^ is similar to that of Ni^2+^, the effective ionic radii of Ni^2+^ is closer to that of Zn^2+^ which is 0.69 Å and 0.74 Å respectively which makes the possibility of substituting Zn^2+^ in ZnO lattice by Ni^2+^. The substitution of Zn^2+^ by Ni^2+^ facilitates the charge separation and transport in the ZnO nanoparticle. Many researches using different elaboration method, demonstrate that the response speed of ZnO-based photodetectors can be improved by using dopant elements without any degradation of the responsivity.^1,16–19^

The polyol method can improve the surface properties of the oxide semiconductors, which could enhance the ability of the oxygen adsorption and desorption.^20^ This method is a low-temperature process that allows higher doping levels of dopant and leads to obtain nanoparticles with high crystalline quality. Additionally, the polyol process may be beneficial for improving the responsivity and reducing the dark current of the devices. In this paper, we report synthesis of Ni doped ZnO nanoparticles using polyol process. Our results indicate that this simple and cost-effective method can be used to improve the photoresponse (response speed, responsivity and dark current) of ZnO based UV PDs. Noise current behavior of the fabricated PDs will also be discussed.

## 2 Experimental details

### 2.1 Preparation of Zn_1−*x*_Ni_*x*_O nanoparticles

Ni-doped ZnO nanoparticles were synthesized according to this method. First, a quantity of zinc acetate dehydrate (Zn(CH_3_COO)_2_·2H_2_O) and Nickel acetate tetrahydrate (Ni(CH_3_COO)_2_·4H_2_O) are well ground in an agate mortar. Then, the powder is added to the sodium hydroxide solution (NaOH) in 80 ml of polyol (1,2-propanediol (PEG), diethylene glycol (DEG)). Thus, the whole is well mixed with ultrasound at room temperature for 30 minutes until the complete dissolution of all the precursors. Finally, a quantity of water is added to reach the hydroxide content and the mixture obtained is heated to the boiling point of the solvent for 6 hours. At the end of the reaction, the synthesized precipitate is centrifuged and washed several times with ethanol and once with acetone and then dried in an oven at 50 °C and then annealed at 300 °C for 1 hour.

**Fig. 1:**
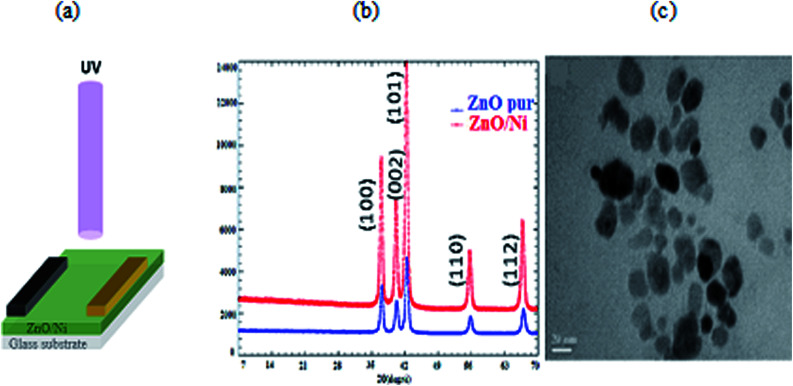
(a) Schematic representation of the fabrication of Ni doped ZnO nanoparticles UV photodetector. (b) XRD pattern of ZnO/Ni nanoparticles. (c) TEM images of Ni doped zinc oxide nanoparticles.

The ZnO/Ni nanoparticles were deposited on glass substrate using spin coating method (2000 rpm for 30 s). Then, an anode (Cu) and indium gallium (InGa) cathode were deposited. The schematic description of the fabrication of UV PD based on Ni doped ZnO nanoparticles is shown in Fig. 1a.

### 2.2 Characterization techniques

The crystalline structure of Ni doped ZnO nanoparticles was characterized by X-ray diffraction (XRD) (a diffractometer using a cobalt radiation of 1.7890 Å). The morphology of the samples was examined by a transmission electron microscopy (TEM) (an JEOL 2011 microscopy operating at 100 kV). The functional groups were determined by a scientific infrared spectrophotometer (Spectrum two FTIR spectrometers by Perkin Elmer), the spectra were recorded in the range 4000–400 cm^−1^.

The optical properties were analyzed by photoluminescence (PL) and diffuse reflectance. The spectra of the diffuse reflectance UV-visible spectra were recorded in the range 200–1000 nm to estimate the energy band gap. Photo-response behavior of PDs was studied using lock-in-amplifier system (SR 830-Stanford Research) with different illumination intensities laser as light source. The noise power spectra were analyzed by PSM 1735 frequency response analyzer.

## 3 Results and discussion

### 3.1 Morphological and structural characterization

The DRX diffraction peaks of pure and Ni doped ZnO NPs is shown in Fig. 1b. The diffraction peaks corresponding to (100), (002), (101), (110) and (112) planes reveal a crystalline hexagonal Wurtzite structure (JCPDS no. 36-1451) and there is no extra peak corresponding to Ni related impurity phases which check that Ni has been incorporated to ZnO interstitial.

In the case of wurtzite phase, the lattice parameters are calculated from the formula:^21^1
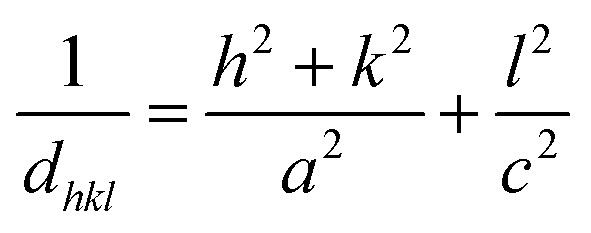
2
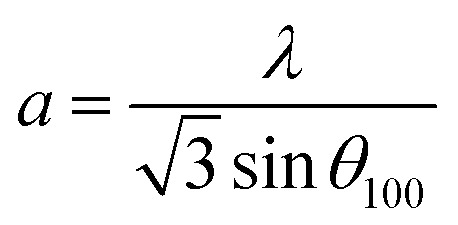
3
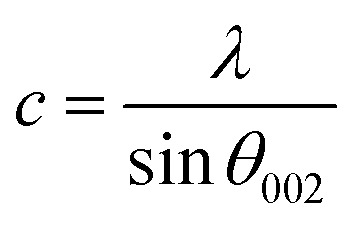

4 *V* = 0.866 × *a*^2^ × *c*

Where *a* and *c* are the lattice parameters, *d*_*hkl*_ is the interplanar distance corresponding to its Miller indices (*hkl*). The structural parameters for the ZnO and Ni doped ZnO are indicated in Table 1. The change in the matrix of the lattice parameter may be due to the presence of lattice strain which slackers after the doping.

**Table 1:** Lattice parameter from the obtained XRD data

Sample	2*θ*	*hkl*	*d*_*hkl*_	Lattice parameter	*c*/*a* ratio
ZnO	31.7321	100	0.2813	*a* = 0.3249	1.603
	34.3951	002	0.2463	*c* = 0.5208	
Ni doped ZnO	31.2421	100	0.2815	*a* = 0.3241	1.602
	33.9104	002	0.2596	*c* = 0.5193	

The calculated lattice parameters of the undoped ZnO are: *a* = 0.3249 nm and *c* = 0.5208 nm; and for the Ni doped ZnO: *a* = 0.3241 nm and *c* = 0.5193 nm. The *D*-spacing for ZnO and Ni doped ZnO is 0.2826 nm and 0.5206 nm, respectively. The values of the volume of the ZnO and Ni doped ZnO nanoparticles are 47.1 and 49.9 nm^3^, respectively (Table 1). We notice that there is a considerable increase in the volume of the crystal structure for the Ni doped ZnO nanoparticles and this may be due to the increase in particle size.^21^

The average crystallite size is given by Debye–Scherrer formula:^21^5
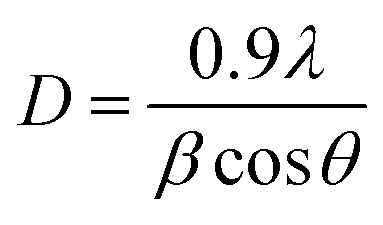
Where *D*, *λ*, *β* and *θ* are crystalline size (in nm), the wavelength of X-ray used in nm, the full width at half maximum (in radian), Bragg diffraction angle (in degree) respectively.

The average calculated crystalline size is in the order of 38 nm for undoped ZnO and 20 nm for Ni doped ZnO NPs.

**Fig. 2:**
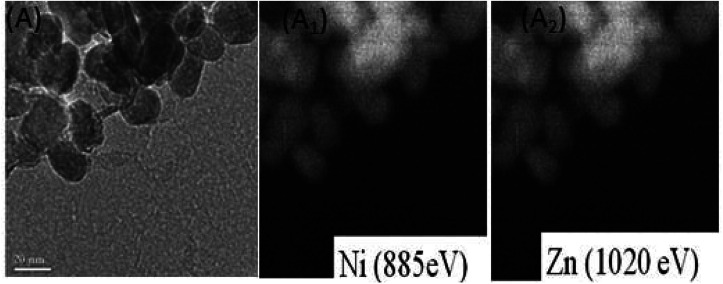
Filter imaging of (A) Ni substituted zinc oxide nanoparticles. It reveals a homogeneous distribution of Ni in ZnO particles (A_1_), filter imaging shows a homogeneous repartition of Zn (A_2_).

To check the occupancy of Ni into the lattice of ZnO, we have chosen most dominant (100), (002) and (101) peaks of undoped and Ni doped ZnO NPs from (Fig. 2). When comparing the diffraction peaks in the range of 2*θ* = 35–45°, we concluded that the peak position of Ni doped ZnO NPs is shifted toward larger 2*θ* value as compared to pure ZnO nanoparticles. This shift is linked to the shrinkage of ZnO lattice due to the substitution by smaller Ni^2+^ (0.055 nm) on Zn^2+^ (0.06) site.^20^

The TEM observation of Ni doped ZnO sample displays almost spherical particles with a size between 20 and 30 nm (Fig. 1c). The distribution of elements in the powders is clarified by selecting and imaging the electrons with a specific energy loss. Analysis was driven on assemblies of particles (Fig. 2) at several regions of Ni-doped ZnO sample. It reveals a homogeneous distribution of Ni in the ZnO particles with no evidence of Ni clusters (Fig. 2A_1_). Concomitantly, imaging analysis shows that the distribution of Zn is also homogeneous for nickel substituted zinc oxides (Fig. 2A_2_).

### 3.2 Optical properties

**Fig. 3:**
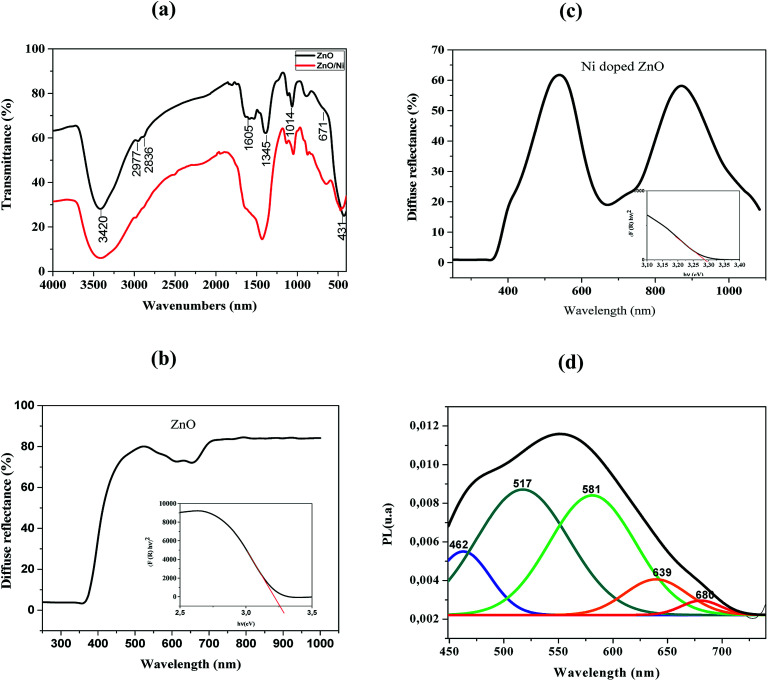
(a) FTIR spectra of ZnO and Ni doped ZnO. (b) The diffuse reflection spectra of ZnO nanoparticles and the Band gap of ZnO pure. (c) The diffuse reflection spectra of Ni doped ZnO and the Band gap of Ni doped ZnO. (d) Photoluminescence spectra of Ni doped ZnO.

FTIR spectroscopy is used to analyze bands of vibrations due to ZnO bands as well as modifications resulting from nickel doping. FTIR spectra of pure ZnO and Ni doped ZnO nanoparticles are recorded in the range between 4000–400 cm^−1^ and are shown in Fig. 3a.

The band around 3420 cm^−1^ and the peak at 1605 cm^−1^ correspond to O–H stretching and bending vibrations of water molecules respectively.^22^ It may be noted that the spectrum of the nickel-doped sample shows a decrease in the intensity of the O–H band relative to the pure ZnO, this can be attributed to the high electronegative power of the Ni^2+^ dopant relative to the hydrogen ions. Ni^2+^ ions were able to break the O–H bond while forming a Ni–O complex that is why the peak has decreased in a remarkable way. The existence of CO_2_ is defined by a peak at 2800 cm^−1^ due to stretching O=C=O vibrations.^23^ The band at 2977 cm^−1^ was attributed to stretching C–H vibration.^24^ The band around 1345 cm^−1^ is attributed to the presence of NO_3_. The peaks appearing between 450 and 600 cm^−1^ indicate the existence of stretching Zn–O vibration.^25^ The vibration modes at 671 and 1014 cm^−1^ are attributed to Ni^2+^ occupation at Zn^2+^ sites.

These results showed some changes compared to pure ZnO. Indeed, the existence of a shift in the frequencies between the two spectra may be due to the difference between the ionic radii of Zn^2+^ and Ni^2+^, which facilitates the substitution of Zn^2+^ by Ni^2+^. This also confirms the incorporation of Ni^2+^ in the ZnO lattice.^25^

Fig. 3b and d illustrate the typical reflectance spectra of the ZnO and Ni doped ZnO nanoparticles with band edge between 375–400 nm. The diffuse reflectance spectrum shows a decrease between 600 nm and 800 nm for Ni doped ZnO nanoparticles. This may be due to intermediate states that are in the band gap. The diffuse reflectance may be very useful in estimating the optical ban gap which may be determined from Kubelka–Munk function *F*(*R*) expressed in terms of reflectance (*R*) using the following equation.7
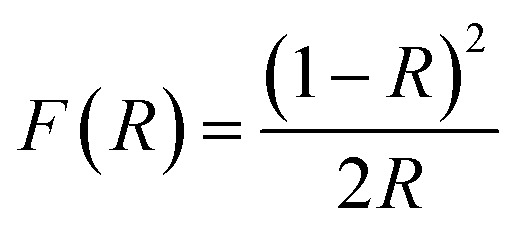


The optical band gap was calculated using Tauc relation as in equation

8 *F*(*R*)*hν* = *A*(*hν* − *E*_g_)^*n*^

Where *n* = 1/2 or 2 are used for direct and indirect transitions, respectively.

Fig. 3c and e represent the plots of (*F*(*R*)*hν*)^2^*versus hν* for undoped and Ni-doped ZnO nanoparticles.

The linear region of these plots was extrapolated to (*F*(*R*)*hν*)^2^ = 0 to get the value of the direct band gap. It is a standard procedure where the linear vertical curve is extrapolated to intercepts the energy of the band gap energy. It is observed that the band gap increases from 3.26 eV of pure ZnO to 3.28 eV of Ni doped ZnO nanoparticles. This may be assigned to sp–d exchange interactions taking place between the band electrons and localized spin of transition metal ions.^26^ In addition, the increase in band gap may be due to an increase in the carrier density which begets a shift of Fermi level close to the conduction band (Burstein–Moss effect).^27^

The PL spectrum of Ni-doped ZnO nanoparticles is shown in Fig. 3f. This spectrum has a broad emission in the visible region. These emissions are generally attributed to structural defects and impurities in the sample.^28–30^

A series of emission bands was observed for Ni doped ZnO nanoparticles; in blue at about 462 nm, in blue-green at about 517 nm, in green at about 581 nm and red at about 639 nm and 680 nm.

Blue emission can be caused by intrinsic defects or/and impurities of nickel. Mainly donor-type defects are zinc interstitials or oxygen vacancies, and acceptor-type defects may be related to zinc vacancies or oxygen interstitial. The blue emission at 462 nm arises to the transition between shallow donor levels and shallow acceptor levels.^31^ The blue-green emission (517 nm) is attributed to the transition from the deep donor levels of the zinc interstitial to the acceptor levels of the zinc neutral. The green emission located at 581 nm is due to doubly ionized oxygen vacancies.^32^

Peaks around 639 nm and 680 nm have been attributed to electron recombination from the donor level associated with the oxygen vacancies to the acceptor level related to impurities, which are due to doping.^28^

### 3.3 *I*–*V*–*t* characteristics of the Ni doped ZnO Nps UV photodetector

The current–voltage (*I*–*V*) characteristics were widely used to investigate the performance of Schottky contacts. The current flowing through the junction is given by the relations:^33^9
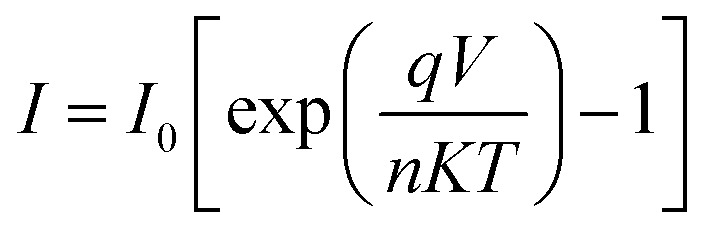
10
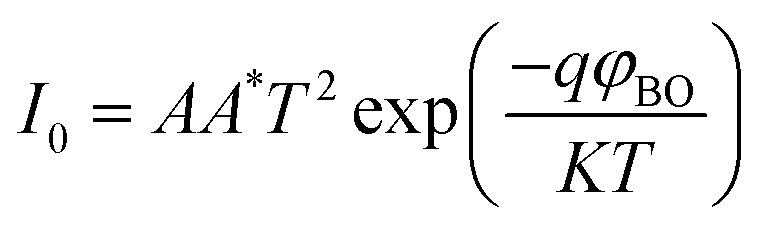
Where *I* is the forward current, *I*_0_ is the saturation current, *q* is the electron charge, *V* is the forward voltage, *n* is the ideality factor, *K* is the Boltzmann constant, *T* is the temperature in kelvin, *A* is the junction area, *φ*_BO_ is the Schottky barrier height and A* is the effective Richardson constant (32 A cm^−2^ K^−2^ for ZnO). By plotting ln *I versus V*, the slope gives 
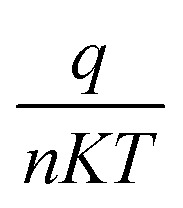
 and the intercept gives ln *I*_0_.

The ideality factor (*n*) can be calculated from the slope of region of the forward bias ln(*I*)–*V* characteristic as^34^11
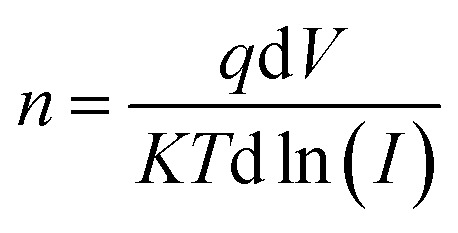


For the ideal diode *n* = 1, but for real diode structure, it is usually greater than unity. Generally, the ideality factor is a parameter indicating the Schottky barrier uniformity. Besides, the effective barrier height *φ*_BO_ can be determined from eqn (10) as12
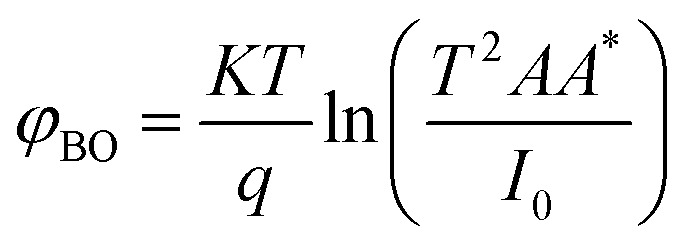


**Fig. 4:**
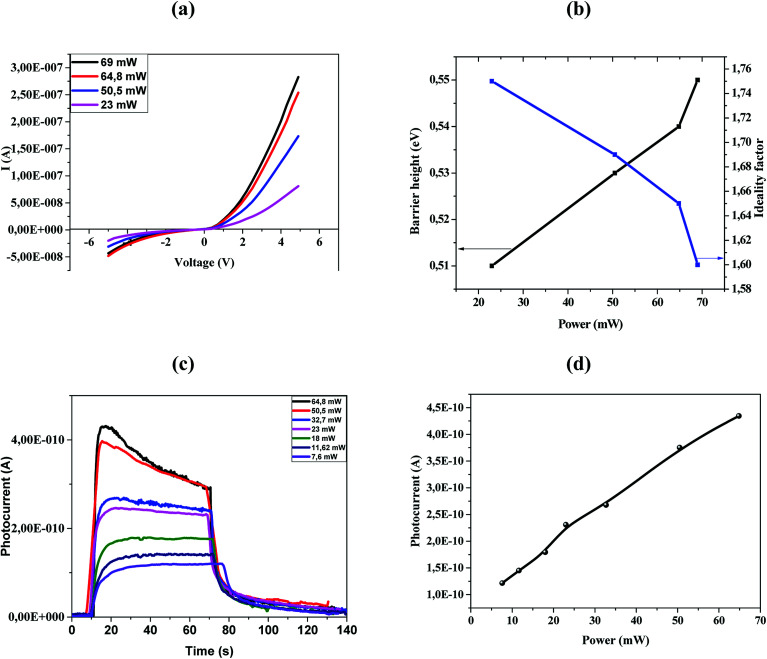
(a) *I*–*V* characteristics of our device at different power density. (b) Barrier height and ideality factor *versus* power. (c) Current of Ni doped ZnO with different UV power densities. (d) Dependence of photocurrent on incident optical power density for 375 nm wavelength.

Fig. 4a shows the *I*–*V* characteristics of the fabricated ZnO/Ni nanoparticles photodetectors under different power.

As shown in Fig. 4a the Schottky contacts exhibit quite good diode behavior for all different power density. The values of the ideality factor (*n*) and barrier height (*φ*_BO_) were calculated from the forward *I*–*V* characteristics, showed in (Fig. 4a). Fig. 4b shows the variations of *n* and *φ*_BO_ with power density. As expected, while *φ*_BO_ increase with an increase in power density, *n* decrease. Different from many previous reports, our device has an excellent stable Schottky barrier height which does not disappear under UV light.

The photocurrent (*I*–*t*) was measured at room temperature in the dark and under illuminated conditions at 375 nm laser light with different incident optical power. As shown in Fig. 4c, the appearance of the pulses reflects the photosensitivity of the material. Under-illumination, the photocurrent is obviously improved when the power density increases. It's clear that the response and recovery time increases with the increase of the light intensity as indicated in Fig. 4c, which are in accordance with others results.^13,35^ The relationship between maximum photocurrent and light intensity is indicated in Fig. 4d. The saturation photocurrent increases linearly with the increasing light intensity.

**Fig. 5:**
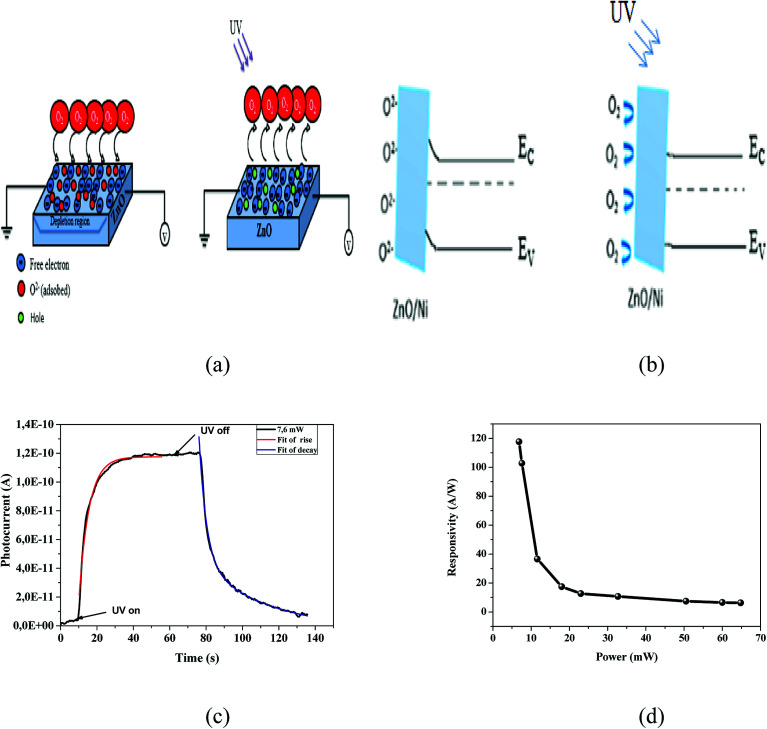
(a) The mechanism of photodetection in the dark and under UV irradiation. (b) The diagram of the depletion zone in Ni doped ZnO in the dark and under illumination. (c) Current rise and decay behavior for Ni doped ZnO under a power of 7.6 mW. (d) Responsivity as functions of the light power intensity at a wavelength of 375 nm.

From the results previously obtained, we can propose the following mechanism (Fig. 5a). The ambient oxygen molecules are adsorbed by capturing free electrons from the n-type surface [O_2_ + e^−^ → O_2_^−^] of Ni doped ZnO nanoparticles. Indeed, a low conductive depletion layer is formed into the surface. Under UV illumination, electron–hole pairs are generated. On the one hand, the generation of electron–hole pairs causes a band-to-band transition process, which explains the rapid rise of the photocurrent in the first few seconds. On the other hand, the photogenerated holes migrate to the surface along the bending band by releasing the oxygen ions that are physically adsorbed [h^+^ + O_2_^−^ → O_2_], which causes the decrease of the depletion layer (Fig. 5b). This process also increases the current flow through the sample.^36–39^

To determine the rise and decay time constants, one cycle of UV illumination under power intensity of 7.6 mW was fitted (Fig. 5c) using bi-exponential expression of the form.^35^

13 *I* = *I*_0_ + *A* e^*t*/*τ*_on_^

14 *I* = *I*_0_ + *B* e^−*t*/*τ*_off_^

Where *τ*_on_ and *τ*_off_ represent the relaxation time constants, whereas *I*_0_, *A*, *B* are positive constants.

The values of the rise and fall time are respectively in the order of 5.52 s and 12 s. The carrier relaxation phenomenon shows two electron processes: the electron trapping by the surface states and electron loss for the recombination at the deep defect states.^39^ From the Fig. 5c; it is indicating that the photodetector shows relatively larger recovery time as compared with rise time. The reason behind the fast rise time is due to the photogenerated carriers undergoing direct band-to-band transition and the longer fall time is due the adsorption of oxygen molecules as increase the depletion layer. The obtained values are lower than those previously reported by many authors. R. Anitha *et al.*^14^ using ZnO microstructure on etched Si:GaN layers, they found a 40 s and 300 s for rise time and decay time respectively. A rise time of 16 s and a decay time of 41 s were reported for carbon nanotube impregnated zinc oxide which fabricated by chemical precipitation process.^36^ The rise time and decay time for ZnO film determined by S. K. Shaikh^2^ were 20 s and 28 s respectively. Also, a rise time of 28 s and the recovery time of 120 s were reported for Mn doped ZnO by Ravishankar Sugumar *et al.*^1^

The performance of the photodetectors can be evaluated by the following relation:^35^15
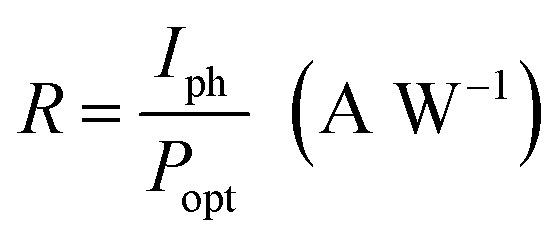
Which *R* is responsivity, *I*_ph_ is photocurrent and *P*_opt_ present the illumination power on the active area of the device.

The responsivity of Ni doped ZnO nanoparticles with a 375 nm UV light excitation as a function of power intensity is given in (Fig. 5d). There is a decrease in the responsivity as a function of the power. This behavior has been found by several works.^37,40,41^

To evaluate the photoresponse performance of the UV photodetector, the photoresponse factor (*S*) is defined as:^35^16
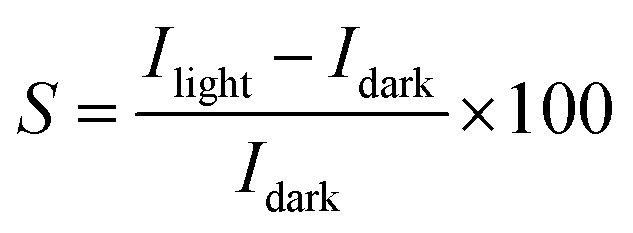


The determined sensitivity is about 4.9 × 10^4^. It is interesting to know that the obtained sensitivity value is higher compared to the value founded by Alsultany (7.5 × 10^3^) using ZnO nanostructures photodetectors.^42^ Also, the value found in our works is significantly higher than the previously reported by Al-Asadi *et al.*^35^ Likewise, a sensitivity of 1154.4 was reported for ZnO nanowire by Ravishankar Sugumar.^1^ Our measurements indicate that the Ni doped ZnO nanoparticles have a much better UV detection performance than the other kind of ZnO nanostructures or films. Thus, Ni doped ZnO nanoparticles have a high surface area/volume ratio that can adsorb oxygen molecules by capturing free electrons. This will leads to ultrahigh photoresponse.

The current gain (*G*) is defined as the ratio between the numbers of absorbed photons to generate photoelectron per unit time and the number of electrons collected per unit time as shown in the following relation:^42^17
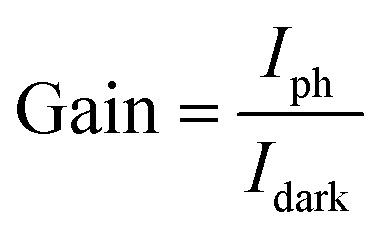


In this work, the current gain (*G*) was 382.4 under a 375 nm wavelength and at a bias voltage of 1 V.

Detectivity is important parameter in evaluating the ability of a photodetectors to detect a weak signal and is another important clue used to characterize photodetectors performance.

The specific detectivity (D*) is calculated through the following relation:^35^18
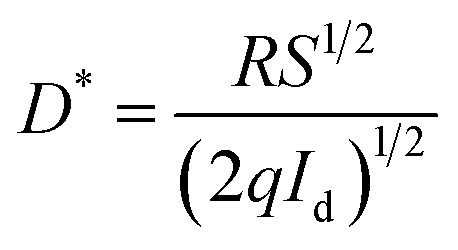
Where *R*, *S*, *q* and *I*_d_ are the responsivity, effective area of illumination of light, electronic charge and dark current respectively. Using experimental data, we found detectivity in the order of 3.7 × 10^10^ jones. However, R. Anitha *et al.*^14^ reported a detectivity of 4.87 × 10^9^ jones, for zinc oxide microstructures on etched Si:GaN layers. Similarly, a detectivity of 1.6 × 10^10^ jones was reported for Mn doped ZnO films.^1^

**Fig. 6:**
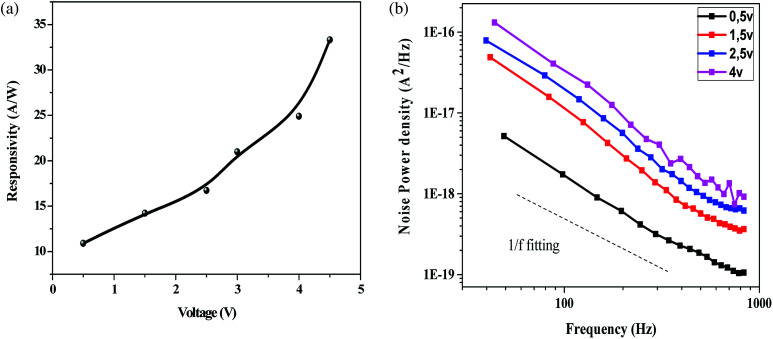
(a) Responsivity spectra of ZnO/Ni nanoparticles based UV photodetector under various voltages biases. (b) Measured low frequency noise power spectra of the photodetector operated under various applied biases.

Fig. 6a indicates the change in responsivity of Ni doped ZnO nanoparticles based UV photodetectors with change in applied voltage. As seen in the plot, the responsivity increases with an increase in bias voltage. For the responsivity performance, the stability Schottky barriers height play an important role.^43^ Also, the existence of strong depletion width could make influence in the responsivity improvement. It can be observed that the maximum responsivity of the device is 300 mA W^−1^ at 4.5 V applied bias. This value of responsivity is larger than the value reported by Pei^44^ and as comparable to ZnO PDs reported by other groups.^43,45^ P. S. Shewale *et al.*^46^ reported the highest value of responsivity for Ti doped ZnO PDs about 50 mA W^−1^.

Our photocurrent measurements allowed us to determine the quantum efficiency that corresponds to the number of electron–hole pairs generated by incident photon, it expressed by:^35^19
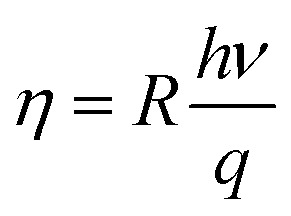
Where *R*, *h*, *ν* and *q* are respectively the responsivity, Planck's constant, the frequency and the charge of the electron. The quantum efficiency deduced from the above equation is of the order of 99.2%. This value is higher as compared to the quantum reported by other groups.^47–49^

Fig. 6b shows the measured low frequency noise power spectra of ZnO/Ni Nps photodetector operating under various applied bias. During noise measurement, the bias voltage was varied from 0.5 to 4 V and the sample was exposed in UV illumination. The resulting spectra density of the noise power against the frequency could be fitted reasonably well with the following Hooge-type equation:^22^20
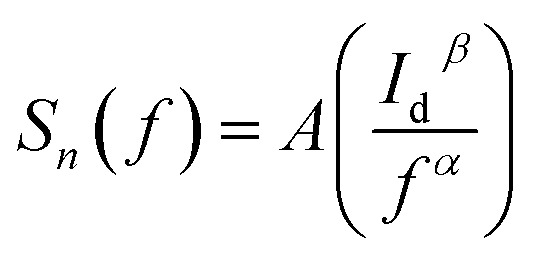
Where *S*_*n*_(*f*) is the spectral density of the noise power, *A* is a constant, *I*_d_ is dark current, *f* is the frequency and *β* and *α* are the two fitting parameters. From the measured curves, the *α* value was nearly unity throughout the measured frequency range implying uniformity of the energy distribution of trapping states.^50^

In generally, the 1/*f* noise was caused by acoustic phonons and ionized defect, which produced mobility fluctuation through impurity scattering and lattice scattering.^50^ Thus, many impurities, such as interstitial zinc and oxygen vacancies presented on our sample can affected the 1/*f* noise spectrum.

The NEP is another important parameters for photodetectors. It represents the minimum optical power that a photodetector can distinguish from the noise.

The total noise current power of the PD can be estimated by integrating *S*_*n*_(*f*) for a given band width *B*:^22^
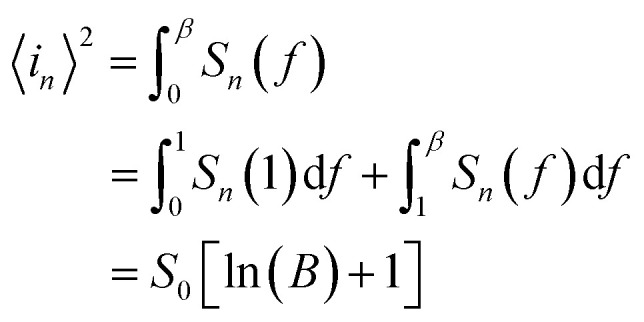


Here, we assume that *S*_*n*_(*f*) = *S*_*n*_(1 Hz),^50^ thus, NEP can be obtained by the following equation:^51^21
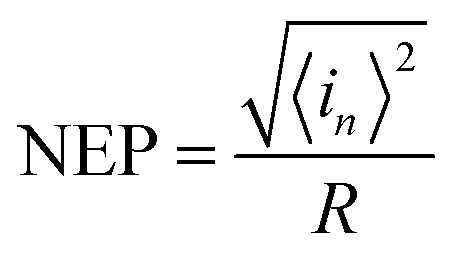
Where *R* is the responsivity of PD.

For a given bandwidth of 1 kHz and a given bias of 4 V, NEP is 1.08 × 10^−9^ W.

Table 2 shows the collected data of our Ni doped ZnO sample as compared to results found in the literature.

**Table 2:** Comparison of the most important UV photodetector parameters between our wok and others study

Materials	Wavelength (nm)	Responsivity [mA W^−1^]	External quantum efficiency (EQE) [%]	Rise time (s)	Decay time (s)	Ref.
ZnSe	—	3.3 × 10^5^	—	10	15	52
ZnO nanofibers	365	—	—	100	50	53
K_2_Nb_8_O_21_	320	2530	98.2	0.3	0.3	47
Ag doped ZnO	365	—	—	1.09	5	54
Graphene oxide	1550	4	0.3	2	2.3	55
ZnO nanowire	365	4	0.3	2	2.3	48
SnO_2_	325	—	—	>40	>40	56
Nb_2_O_5_	320	1520	60.7	4	9	49
InSe	633	3.9 × 10^3^	69	0.04	4	57
ZnO nanowire	355	7.5	—	5	6.7	58
Al doped ZnO	365	0.031	—	0.7	2.5	59
Ni doped ZnO NPs	375	1.21 × 10^5^	92	5.2	12	This work

The performances of our devices indicate the possibility of using Ni doped ZnO nanoparticles to develop photodetectors that demonstrate improved performance using a simple, and low cost elaboration method.

## 4 Conclusion

In summary, Ni doped ZnO nanoparticles are designed for UV photodetector by using simple polyol method. The fabricated Ni doped ZnO nanoparticles UV photodetector exhibits ultrahigh photoresponse with fast photoswitching characteristics having rise time about 5.52 s. Owing to large area to volume ratio for chemisorptions of oxygen molecules, fabricated photodetector shows sensitivity about 4.89 × 10^4^ at 375 nm wavelength. Photocurrent measurements confirm that a morphological and structural property significantly affects photoresponse properties of the photodetector. The Ni doped ZnO nanoparticles photodetector offers high responsivity, fast response time, low noise current and room temperature operating functionality. These results pave the way for utilizing Ni doped ZnO nanoparticles-based UV photodetector in the development of fast switching optoelectronic devices.

## Conflicts of interest

There are no conflicts to declare.

## Supplementary Material
